# Protocol update to: High-throughput scNMT protocol for multiomics profiling of single cells from mouse brain and pancreatic organoids

**DOI:** 10.1016/j.xpro.2025.103980

**Published:** 2025-07-24

**Authors:** Santiago Cerrizuela, Oguzhan Kaya, Lukas P.M. Kremer, Andrea Sarvari, Tobias Ellinger, Jannes Straub, Jan Brunken, Andrés Sanz-Morejón, Aylin Korkmaz, Ana Martín-Villalba

**Affiliations:** 1 Molecular Neurobiology, German Cancer Research Center (DKFZ), 69120 Heidelberg, Germany

**Keywords:** Bioinformatics, Sequence analysis, Cell Biology, Single Cell, Genomics, RNA-seq, Molecular Biology, Neuroscience

## Abstract

Single-cell nucleosome, methylome, and transcriptome (scNMT) sequencing is a recently developed method that allows multiomics profiling of single cells. In this scNMT protocol, we describe profiling of cells from mouse brain and pancreatic organoids, using liquid handling platforms to increase throughput from 96-well to 384-well plate format. Our approach miniaturizes reaction volumes and incorporates the latest Smart-seq3 protocol to obtain higher numbers of detected genes and genomic DNA (gDNA) CpGs per cell. We outline normalization steps to optimally distribute per-cell sequencing depth.

For complete details on the use and execution of this protocol, please refer to Kremer et al. and other works.^1^^,^^2^^,^^3^^,^^4^^,^^5^^,^^6^^,^^7^

This protocol is an update to Cerrizuela et al.^7^

## Highlights

• Throughput increase to 384-well plate format for methylome with a decrease in costs
• Validation of the protocol in two research papers
• Improved troubleshooting for optimization of cDNA quality
• Incorporation of patterned-flow cells for sequencing of transcriptomic libraries

## Abstract (editor highlights)

Publisher’s note: Undertaking any experimental protocol requires adherence to local institutional guidelines for laboratory safety and ethics.

## Graphical abstract

**Figure:**
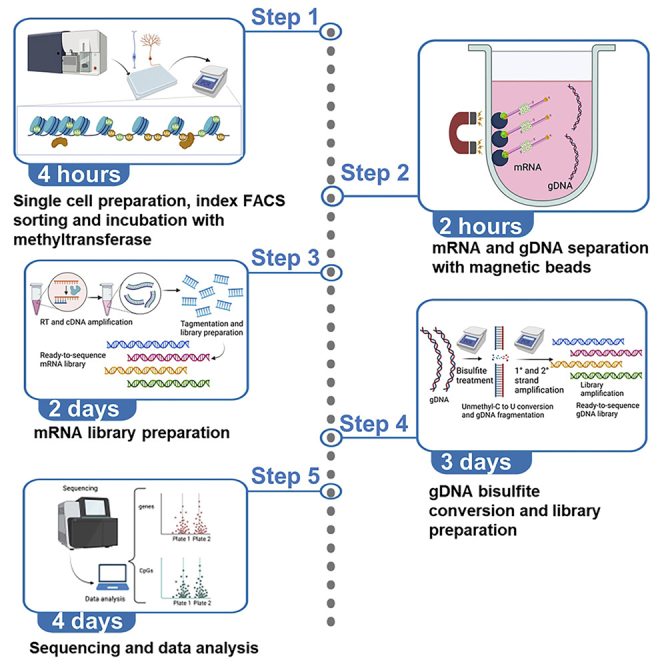

## Before you begin

The profiling of mRNA species, chromatin accessibility and epigenetic information in single cells is key to understanding the molecular mechanisms governing how fate choices are executed within a cell. In this regard, single cell sequencing methods underwent an exciting development in the last years, with the incorporation of multiomics profiling, which has the advantage of capturing multiple molecular layers from the same cell.^8^ Recently, the scNMT protocol was used to capture the transcriptomic and epigenetic landscape of mouse gastrulation.^4^^,^^9^ However, the protocol is cost-intensive, uses Smart-seq2 as the RNA sequencing pipeline and works on a 96-well plate format.

Due to tissue heterogeneity and the stochasticity of states present even within the very same cell type, it is important to profile a significant number of cells to have statistically relevant readouts and address the biology of a tissue.

This would allow a comprehensive understanding of the regulatory mechanisms underlying transcriptional commitment and cell fate decisions, together with the discovery of key molecular drivers and mechanisms underpinning cellular states in homeostasis and disease. Ultimately, precise tuning of epigenetic states may permit greater control of engineered cell type and activity for cell therapies.

Here, we improve the scNMT protocol by incorporating the Smart-seq3^5^^,^^6^ pipeline for the transcriptomic profiling, which uses unique molecular identifiers (UMIs) to pre-label each unique molecule, addressing potential bias introduced during amplification and enabling the quantitative measurement of RNA molecules. Moreover, the implementation of Smart-seq3 allows capturing a much higher number of genes per cell, including transcription factors, which are very important to regulate chromatin and are generally lowly expressed. In addition, we miniaturize the reaction volume and increase the throughput to a 384-well plate format, with the possibility of profiling up to 768 cells per sequencing run (at the mRNA level and the gDNA level). To improve the number of cells that reach the lower threshold of genes/cell, we also incorporate normalization steps in order to optimally distribute sequencing depth among all the cells.

For an optimal protocol, we recommend the use of automatic pipetting machines, such as Viaflo 384 (Integra), Mosquito HV (SPT Labtech) and Mantis (FORMULATRIX). In the absence of these machines, a multichannel pipette can be utilized, which would increase the plate-processing time.

The protocol below describes the specific steps for profiling mouse primary neural stem cells (NSCs), astrocytes, neuroblasts, oligodendrocytes and neurons, as well as cells from pancreatic acinar organoids.

As mentioned before, we have increased now the throughput to be 384-well for the mRNA as well as for the gDNA part, which decreases further the sequencing costs. This protocol was used and validated on the following papers.^1^^,^^2^

### Institutional permissions

All animal experiments were performed in accordance with the institutional guidelines of the DKFZ and were approved by the “Regierungspräsidium Karlsruhe” (Germany).

## Key resources table

**Table:** 

REAGENT or RESOURCE	SOURCE	IDENTIFIER
**Chemicals, peptides, and recombinant proteins**		

Buffer RLT Plus	QIAGEN	Cat#1053393
Dynabeads MyOne streptavidin C1	Invitrogen	Cat#65001
10 mM dNTP mix	Thermo Scientific	Cat#R0192
1 M DTT	Sigma-Aldrich	Cat#43816-10 mL
KAPA HiFi HotStart ReadyMix (2×)	Roche Diagnostics	Cat#KK2602/07958935001
KAPA HiFI HotStart PCR mix	Roche Diagnostics	Cat#KK2502/07958897001
CT conversion reagent	Zymo Research	Cat#D5003-1
M-solubilization buffer	Zymo Research	Cat#D5021-7
M-dilution buffer	Zymo Research	Cat#D5002-2
M-reaction buffer	Zymo Research	Cat#D5021-8
MagBinding beads	Zymo Research	Cat#D4100-2-8
M-binding buffer	Zymo Research	Cat#D5002-3
M-desulfonation buffer	Zymo Research	Cat#D5001-5
Klenow (3′–5′ exo-)	Biozym	Cat#280310
Exonuclease I	NEB	Cat#M0293L
Recombinant RNase inhibitor	Takara Bio	Cat#2313A
Agencourt AMPure XP beads	Beckman Coulter	Cat#A63881
Maxima H Minus	Thermo Scientific	Cat#EP0752
Nextera XT DNA sample preparation kit	Illumina	Cat#FC-131-1096
IGEPAL CA-630	Sigma-Aldrich	Cat#I8896-50mL
1 M Tris-HCl buffer pH 8.0	Thermo Fisher Scientific	Cat#15568025
0.5 M EDTA solution	Thermo Fisher Scientific	Cat#15575020
Tween 20	Sigma-Aldrich	Cat#P1379-1L
SYTOX Blue dead cell stain	Thermo Scientific	Cat#S34857
TrypLE Express enzyme (1×), no phenol red	Thermo Scientific	Cat#12604013
PEG 8000	Sigma-Aldrich	Cat#P2139

**Critical commercial assays**		

Bioanalyzer DNA high sensitivity chip	Agilent	Cat#5067-4626
Qubit assay tubes	Thermo Fisher Scientific	Cat#Q32856
Qubit dsDNA HS assay kit	Thermo Fisher Scientific	Cat#Q32854
Quant-iT PicoGreen dsDNA assay kits	Thermo Fisher Scientific	Cat#P7589
Neural tissue dissociation kit (NTDK)	Miltenyi Biotec	Cat#130-093-231

**Experimental models: Cell lines**		

Primary cells from mouse brain	JANVIER LABS	C57BL/6NRj
Mouse pancreatic organoids	JANVIER LABS	C57BL/6NRj

**Oligonucleotides**		

Biotinylated Oligo-dT_SS3 /5BiotinTEG/	This paper	AC GAG CAT CAG CAG CAT ACG ATT TTT TTT TTT TTT TTT TTT TTT TTT TTT TVN
Template-switching oligo (TSO) /5Me-isodC//iisodG//iMe-isodC/	This paper	AGA GAC AGA TTG CGC AAT GNN NNN NNN NNN NrGrG+G
mRNA index primers – combinatorial indexing – 5er and 7er	Nextera index primers. For the original design see, Buenrostro et al.^10^ in the references of the manuscript.	On Table S1
Fwd_PCR_primer	Hagemann-Jensen et al.,^5^^,^^6^ see references on manuscript	TCGTCGGCAGCGTCA GATGTGTATAAGAG ACAGATTGCGCAA∗T∗G
Rev_PCR_primer	Hagemann-Jensen et al.,^5^^,^^6^ see references on manuscript	ACGAGCATCAGCAGCATAC∗G∗A
Preamp oligo	Clark et al.,^4^ see references on manuscript	CTACACGACGCTCT TCCGATCTNNNNNN
Adapter 2 oligo	Clark et al.,^4^ see references on manuscript	TGCTGAACCGCTCTTC CGATCTNNNNNN
gDNA index primers – Unique dual indexing	This paper	On Table S2
iTAG sequencing primer	Clark et al.,^4^ see references on manuscript	AAGAGCGGTTCAGCAGGA ATGCCGAGACCGATCTC

**Software and algorithms**		

zUMIs	Parekh et al.,^11^ see references on manuscript.	https://github.com/sdparekh/zUMIs
FASTQC	Andrews et al.,^12^ see references on manuscript.	https://www.bioinformatics.babraham.ac.uk/projects/fastqc/
STAR mapper 2.7.3a	Dobin et al.,^13^ see references on manuscript.	https://github.com/alexdobin/STAR
Trim Galore	Krueger,^14^ see references on manuscript.	https://www.bioinformatics.babraham.ac.uk/projects/trim_galore/
Bismark	Krueger & Andrews,^15^ see references on manuscript.	https://www.bioinformatics.babraham.ac.uk/projects/bismark/
BioRender	Science Suite Inc.	https://biorender.com/
Inkscape	The Inkscape Project	https://inkscape.org/

**Other**		

twin.tec PCR plate 384 LoBind	Eppendorf	Cat#00301129547
Hard-Shell 384-well PCR plates, thin wall, skirted, clear/clear	Bio-Rad	Cat#HSP3801
5 XYZ racks of 384 tips	INTEGRA	Cat#6465
384-well plate magnetic block	SPT Labtech	Cat#3268-02008
neoVortex shaker, with fixed rotation 2500 rpm	neoLab	Cat#D-6013
Spool of 18500 gamma ray sterilized pipettes 4.5 mm Pitch HV	SPT Labtech	Cat#4150-03033
VIAFLO 384 base unit	INTEGRA	Cat#6031
384-channel pipetting head 2–50 μL	INTEGRA	Cat#6136
16 channel VIAFLO lightweight electronic pipette	INTEGRA	Cat#4646
Mantis V3.3 with integrated RFID	FORMULATRIX	Cat#MANTV3.3_RFID
MANTIS chip – silicone, LV (0.1 μl & 0.5 μl), RFID	FORMULATRIX	Cat#233649
MANTIS chip – silicone, HV (1 μl & 5 μl), RFID	FORMULATRIX	Cat#233648
MANTIS chip – silicone, continuous Flow (CF), RFID	FORMULATRIX	Cat#232724
Mosquito HV liquid handling system	SPT Labtech	Cat#3097-01057
NextSeq 2000 sequencing system	Illumina	Cat#20038897
NextSeq 550 sequencing system	Illumina	Cat#SY-415-1002
NextSeq 1000/2000 P2 reagents (200 cycles) v.3	Illumina	Cat#20046812
NextSeq 500/550 mid output Kit v.2.5 (150 CYS)	Illumina	Cat#20024904
NextSeq 500/550 High Output Kit v.2.5 (150 CYS)	Illumina	Cat#20024907

^∗^Phosphorothioate.

## Materials and equipment

As described before, we recommend liquid handler platforms to obtain reproducible results. Viaflo 384 (INTEGRA) can be used for the nucleic acid purification steps and gDNA and mRNA separation. Mantis (FORMULATRIX) is used for adding low amounts of cost-intensive reagents with little dead volume. Viaflo 16-channel electronic pipette (INTEGRA) is used to prepare pre-aliquoted plates with master mixtures or reagents.

For dispensing low volumes (less than 3 μL) we usually use Mantis (FORMULATRIX) and Mosquito HV (SPT Labtech), while for dispensing larger volumes we pre-aliquot the specific solution into a fresh 384-deep well plate and then use Viaflo 384 (INTEGRA) to transfer it. For aspirating lower volumes, we use Mosquito HV (SPT Labtech) and for higher volumes we use Viaflo 384 (INTEGRA). When proceeding with the protocol, it is up to the user to decide how to dispense and aspirate solutions.

Of note, the 384-well plates should be DNA low-bind and PCR-clean. We opted for twin.tec PCR Plates LoBind (Eppendorf, see key resources table), but Hard-Shell 384-well PCR Plates (Bio-Rad) worked equally well based on our experience.

Below we describe the solutions that need to be prepared in order to perform the protocol.

### Transcriptomic part

#### Oligo dT bead preparation

**Timing: 20 min**

**Table:** Dynabead solution A

Reagent	Final concentration	Amount
NaOH (10 M)	0.1 M	100 μL
NaCl (5 M)	0.05 M	100 μL
ddH_2_O	N/A	9.8 mL
**Total**	N/A	**10 mL**

Can be stored at 4°C for 1 month.

**Table:** Dynabead solution B

Reagent	Final concentration	Amount
NaCl (5 M)	0.1 M	200 μL
ddH_2_O	N/A	9.8 mL
**Total**	N/A	**10 mL**

Can be stored at 4°C for 1 month.

**Table:** Dynabead 2× “Bind and wash” buffer (B&W)

Reagent	Final concentration	Amount
Tris-HCl pH 7.5 (1 M)	0.01 M	100 μL
EDTA (0.5 M)	1 mM	20 μL
NaCl (5 M)	2 M	4 mL
ddH_2_O	N/A	5.88 mL
**Total**	N/A	**10 mL**

Can be stored at 4°C for 1 month.

**Table:** Genome and transcriptome wash buffer (G&T)

Reagent	Final concentration	Amount
Tris-HCl pH 8.3 (1 M)	0.05 M	500 μL
KCl (3 M)	0.075 M	250 μL
MgCl_2_ (1 M)	3 mM	30 μL
DTT (0.1 M)	0.01 M	1 mL
Tween-20 (100%)	0.5%	50 μL
ddH_2_O	N/A	8.17 mL
**Total**	N/A	**10 mL**

Can be stored at 4°C for 1 month.

**Table:** PEG 50%

Reagent	Final concentration	Amount
PEG 8000	50% w/v	5 g
ddH_2_O	N/A	Add until
**Total**	N/A	**10 mL**

Can be stored at 4°C for 6 months. This solution is used It is used for the RT master mix.

### Genomic part

#### Conversion reagent

**Timing: 20 min**

**Table:** 

Reagent	Final concentration	Amount
CT Conversion Reagent (powder)	N/A	1 bottle
M-solubilization buffer	N/A	7.9 mL
M-Dilution buffer	N/A	3 mL
M-Reaction buffer	N/A	1.6 mL
**Total**	N/A	**12.5 mL**

• Add 7.9 mL of M-solubilization Buffer + 3 mL of M-Dilution Buffer into CT Conversion Reagent (Powder bottle). Vortex 10 min.
• Add 1.6 mL of M-Reaction Buffer. Vortex 3 min. Aliquot into dark 1.5 mL microcentrifuge tubes and store for up to 1 month a −20°C. 1 bottle of conversion reagent can be used to process 1 ½ plates.

### PEG buffer 18%

**Timing: 20 min**

**Table:** 

Reagent	Final concentration	Amount
PEG 8000	18% w/v	9 g
NaCl	2.5 M	7.3 g
Tris-HC pH 8 (1 M)	10 mM	500 μL
EDTA (0.5 M)	1 mM	100 μL
100% Tween-20	0.05%	25 μL
ddH_2_O	N/A	Add until 50 mL
**Total**	N/A	**50 mL**

Sterilize through a 0.2 μm filter and treat the buffer with UV irradiation for 1 h (a laminar flow hood with UV light can be used to sterilize the solution). It can be stored for 1 month at 4°C.

## Step-by-step method details

### Tissue processing for the preparation of single-cell suspension

**Timing: 2–3 h**

Here a solution of single cells will be prepared starting from the tissue or cell system of origin.

***Note:*** The time for obtaining a single cell preparation depends on the starting tissue. For isolating neural stem cells, neuroblasts and neurons, the times per mouse are explained below. Subventricular zone and olfactory bulb dissociation: 15 min. Tissue processing and single cell preparation with the Neural Tissue Dissociation Kit (NTDK, Miltenyi, see key resources table): 40 min (follow the manufacturer protocol: https://www.miltenyibiotec.com/upload/assets/IM0001320.PDF). Antibody labeling and final wash: 30 min. For more information on the sorting strategy see the following paper.^16^

For the isolation of single cells from pancreatic organoids the times are explained below. Incubation of organoid with TrypLE Express: 30 min. Washing and single cell resuspension: 30 min. For more information on the generation of pancreatic organoids, see the following papers.^17^^,^^18^

### Single-cell collection and methyltransferase reaction

**Timing: 1 h**

Through this step, cells will be sorted into 384-well plates and will undergo a reaction with a methyltransferase to obtain information about DNA accessibility.

***Note:*** Prepare a single cell suspension of your cell type of interest. Then proceed to cell collection. Collect cells on individual wells of a low binding 384-well plate. For sorting live cells, we use Sytox blue dead cell stain (see key resources table) on our sorting strategy. The use of FACS is preferred. Alternatively, cell printing devices like cellenONE (Cellenion) can also be used. The collection solution is composed of a mild lysis buffer (IGEPAL CA-630), used to permeabilize the nuclear membrane and a GpC methyltransferase (M.CviPI), which adds methyl groups to cytosines in GpC islands located in non-nucleosome bound DNA. The latter allows the assessment of chromatin accessibility throughout the genome.

1. Prepare GpC methyltransferase reaction buffer. Prepare fresh and keep on ice.

**Table:** GpC methylase master mix

Reagent	Final concentration	Amount for 1 reaction	Amount for 1 plate (384 + 10%)
ddH_2_O		0.57 μL	240.79 μL
GpC methyltransferase buffer (10×)	1×	0.1 μL	42.24 μL
SAM (32 mM)	0.16 mM	0.005 μL	2.11 μL
IGEPAL CA-630 (1%)	0.1%	0.1 μL	42.24 μL
RNase-in (40 U/μL)	1 U/μL	0.025 μL	10.56 μL
M.CviPI (4 U/μL)	0.8 U/μL	0.2 μL	84.48 μL
Total		1 μL	422.42 μL

2. Add 1 μL of the mix into every well of a 384-well plate. Index sort single cells on the plate.

***Note:*** Index sorting is a mode of the FACS instrument that allows the isolation of single cells while recording the coordinates of all fluorescence and scatter parameters for each individual event. It is advisable to keep the temperature of the plate holder at 4°C during sorting.

***Optional:*** Sort minibulks of 25–100 cells per cell type into single wells as positive control and to obtain a deeper coverage from both the transcriptome and the genome of specific cell populations. For minibulks, the volume per well and the incubation time can remain the same as with single cells.

**CRITICAL:** The FACS sorter or cell printing device (e.g. FACS Aria II) should have a 4°C cooling block, to keep the methyltransferase reaction inhibited until all the cells are sorted.

3. After sorting, centrifuge the plate at 1,000 × *g* for 10 s.
4. Incubate on a thermocycler for 15 min at 37°C with the lid at 50°C. Add 2 μL of RLT Plus buffer and centrifuge at 1,000 × *g* for 20 s.

**CRITICAL:** Aliquot the RLT Plus buffer upon arrival to avoid contamination of the reagent, which can cause a decrease in the quality of the cDNA library.

**Pause Point:** Store plate at −80°C or proceed directly with the separation of the mRNA and gDNA.

### Separation of mRNA from gDNA

#### Oligo dT bead preparation

**Timing: 40 min**

Here the binding of the dynabeads to the oligo-dT takes place through the strong interaction between Streptavidin and Biotin. To that end, we prepare and wash the dynabeads with a series of buffers.

5. Add 225 μL Dynabeads (MyOne Streptavidin C1) to a 1.5 mL microcentrifuge tube.
  a. Place on a magnet, wait 30 s and remove supernatant (SN).
6. Wash the beads with Solutions A, B and B&W buffer.
  a. Resuspend beads in 400 μL of Solution A by pipetting outside the magnet. Place on magnet, wait 30 s and remove the SN.
  b. Repeat step a one more time.
  c. Resuspend beads in 200 μL of Solution B by pipetting outside the magnet. Place on magnet, wait 30 s and remove the SN.
  d. Resuspend the beads in 225 μL of 2× B&W buffer and add 225 μL of Oligo-dT-SS3 (100 μM).
  e. Incubate by rotation for 15 min at 18°C–25°C.

**Pause Point:** This solution can be stored overnight at 4°C.

7. Prepare the bead resuspension solution, G&T wash buffer + RNase inhibitor and the reverse transcription (RT) Master Mix.

**Table:** Bead resuspension solution

Reagent	Final concentration	Amount for 1 reaction (1×)	Amount for 1 plate (384 + 15%, i.e., 441.6×)
ddH_2_O		3.1 μL	1368.96 μL
Buffer RT (5 ×) from Maxima enzyme	1 ×	0.8 μL	353.28 μL
RNAse Inhibitor (40 U/μL)	1 U/μL	0.1 μL	44.16 μL
Total		4 μL	1766.4 μL

Store on ice until use. Once used to resuspend the Oligo-dT beads, aliquot solution into a fresh 384-well plate.

**Table:** G&T Wash buffer with RNase Inhibitor

Reagent	Final concentration	Amount for 1 reaction (1×)	Amount for 1 plate (384 + 15%, i.e., 441.6×)
G&T Wash buffer		14.85 μL	6557.76 μL
RNase Inhibitor (40 U/μL)	0.4 U/μL	0.15 μL	66.24 μL
Total		15 μL	6624 μL

Store on ice until use.

***Optional:*** Aliquot the G&T wash buffer + RNase Inhibitor in a new plate to increase the efficiency of the separation of mRNA and gDNA. By aliquoting the G&T wash buffer in a different plate, we reduced the time for transferring the wash buffer to the sample plate, thereby decreasing the time that the beads are drying out. Over-drying beads might prevent an efficient elution of the mRNA in the RT mix.

**Table:** RT master mix (mind the order)

Reagent	Initial concentration	Amount for 1 reaction (1×)	Amount for 1 plate (384 + 15%, i.e., 441.6×)	Final concentration
ddH_2_O		1.185 μL	523.30 μL	
dNTP mix	10 mM	0.2 μL	88.32 μL	1 mM
Tris-HCl pH 8.3	1 M	0.040 μL	17.66 μL	25 mM
NaCl	1 M	0.060 μL	26.5 μL	30 mM
MgCl_2_	100 mM	0.050 μL	22.08 μL	2.5 mM
GTP	100 mM	0.020 μL	8.83 μL	1 mM
TSO (Template switching oligo)	100 μM	0.040 μL	17.66 μL	2 μM
RNase Inhibitor	40 U/μL	0.025 μL	11.04 μL	0. 5 U/μL
PEG	50%	0.2 μL	88.32 μL	5%
DTT (add second to last)	100 mM	0.160 μL	70.66 μL	8 mM
Maxima H-minus RT (add last)	200 U/μL	0.020 μL	8.83 μL	2 U/μL
Total		2 μL	883.2 μL	

Store on ice until use.

### Bead resuspension and separation of genomic and transcriptomic fractions

**Timing: 30 min**

Through these steps the polyadenylated RNAs will bind to the oligo-dT-bound dynabeads which will be kept on the well through the use of a magnetic block. On a second step, the gDNA will be separated from the RNA faction by a series of washes.

8. Resuspend the beads in 400 μL of B&W buffer 1× by pipetting outside the magnet.
  a. Place on magnet, wait 30 s and remove the SN.
  b. Repeat this step 3 times more to reach a total of 4 washes.
9. Resuspend beads with the prepared Bead Resuspension solution. Mix by vortexing thoroughly 15 s (we apply 2500 rpm for vortexing).

**CRITICAL:** After resuspending the beads with the bead resuspension solution, please use the solution within the next 30 min, to keep the RNase inhibitor within its optimal activity time window.

10. Transfer 4 μL of bead resuspension solution to the mRNA plate.
  a. Vortex 30 s at maximum speed, spin down the plate and incubate 3 min at 18°C–25°C.
  b. Repeat twice more to reach a total incubation time of approx. 10 min.
  c. After the incubation centrifuge at 1,000 × *g* for 1 min at room temperature (RT, 18°C–25°C).
11. Separation of gDNA from polyadenylated RNAs.
  a. Place the plate on magnet and incubate 2 min until the suspension is clear.
  b. Aspirate 7 μL from the mRNA plate still on the magnet and transfer to the gDNA plate.
  c. Add 5 μL of G&T wash buffer to the mRNA plate.
  d. Vortex mRNA plate for 30 s, spin down on a benchtop centrifuge for 10 s and incubate 1 min.
  e. Repeat step d two more times to achieve an incubation time of 5 min. Centrifuge the mRNA plate for 1 min at 1,000 × *g.*
  f. Put mRNA plate on magnet, incubate 2 min and transfer SN to gDNA plate.
  g. Repeat steps c-f twice more. Now you have 22 μL of gDNA eluate on the gDNA plate. Store the gDNA plate at −20°C until proceeding with the gDNA library preparation at step 38.
12. Immediately add 2 μL of the 18°C–25°C mix to the mRNA plate on ice.

### Transcriptomic part

#### Reverse transcription, cDNA amplification, and cDNA purification

**Timing: 6 h**

Here the polyadenylated RNA will be retrotranscribed, and then subsequently amplified. At the end the cDNA will be purified with magnetic beads.

Reverse transcription and cDNA amplification.

Through these steps the polyA mRNA will be reverse-transcribed and amplified. Afterward, the obtained cDNA will be purified before proceeding with tagmentation and library amplification.

The first PCR is a standard reverse transcription reaction with no amplification step. In the following PCR amplification step, generating a double stranded cDNA from the single stranded cDNA template needs to be optimized per cell type to ensure amplification of just sufficient cDNA with minimal PCR amplification bias. For testing this, we advise users to prepare a so-called validation plate per cell type to run tests for determining RNA quality and optimal PCR cycle before proceeding with a sample plate.

13. Incubate plate on a thermocycler as follows.
  **Table:** Reverse transcription. PCR cycling conditions

Temperature	Time	Cycles
42°C	90 min	1
50°C	2 min	10
42°C	2 min	
85°C	5 min	1
4°C	Hold

  ***Optional:*** A pre-wash of unbound TSO before proceeding to the cDNA amplification step might help reducing TSO dimers (peak between 80 and 150 bp in the Bioanalyzer traces). To avoid this, perform a TSO wash after the reverse transcription. We therefore advise to perform a test run with few samples to see whether TSO dimers represent a problem for the tested samples. If so, proceed with TSO wash.
  a. After reverse transcription, place mRNA plate on magnet and incubate 3 min.
  b. Discard SN. Add 20 μL of 1× RT buffer.
  c. Vortex 30 s and spin down. Incubate on magnet 3 min. Discard SN and add TSO-wash cDNA amplification master mix (5 μL).
    **Table:** TSO-wash cDNA amplification master mix (when doing TSO-wash)

Reagent	Initial concentration	Amount for 1 reaction (1×)	Amount for 1 plate (384 + 15%, i.e., 441.6×)	Final concentration
ddH_2_O		3.075 μL	1357.92 μL	
HiFi HotStart (KAPA HiFI HotStart Polymerase)	1 U/μL	0.1 μL	44.16 μL	0.02 U/μL
5× HiFi Fid. With Mg (KAPA High Fidelity Buffer 5×)	5 ×	1 μL	441.6 μL	1 ×
MgCl_2_	100 mM	0.075 μL	33.12 μL	0.5 mM
dNTP mix	10 mM	0.15 μL	66.24 μL	0.5 mM
Forward Primer	10 μM	0.25 μL	110.4 μL	0.5 μM
Reverse Primer	10 μM	0.05 μL	22.08 μL	0.1 μM
PEG	50%	0.2 μL	88.32 μL	5%
Tris-HCl pH 8.3	1 M	0.040 μL	17.66 μL	25 mM
NaCl	1 M	0.060 μL	26.5 μL	30 mM
Total		5 μL	2208 μL
14. If no TSO wash is performed, prepare cDNA amplification master mix (MM) and add 3 μL of the MM to the plate. Vortex gently and spin down on benchtop centrifuge.
  **Table:** cDNA amplification master mix

Reagent	Initial concentration	Amount for 1 reaction (1×)	Amount for 1 plate (384 + 15%, i.e., 441.6×)	Final concentration
ddH_2_O		1.425 μL	629.28 μL	
HiFi HotStart (KAPA HiFI HotStart Polymerase)	1 U/μL	0.1 μL	44.16 μL	0.02 U/μL
5× HiFi Fid. With Mg (KAPA High Fidelity Buffer 5×)	5 ×	1 μL	441.6 μL	1 ×
MgCl_2_	100 mM	0.025 μL	11.04 μL	0.5 mM
dNTP mix	10 mM	0.15 μL	66.24 μL	0.5 mM
Forward Primer	10 μM	0.25 μL	110.4 μL	0.5 μM
Reverse Primer	10 μM	0.05 μL	22.08 μL	0.1 μM
Total		3 μL	1324.8 μL
  a. Incubate plate on thermocycler as follows:
    **Table:** cDNA Amplification. PCR cycling conditions

Steps	Temperature	Time	Cycles
Initial Denaturation	98°C	3 min	1
Denaturation	98°C	20 s	18–27
Annealing	65°C	30 s	
Extension	72°C	4 min	
Final extension	72°C	5 min	1
Hold	4°C

    ***Note:*** Cycle number depends strongly on the cell type used. In our case we used 24 cycles for primary NSCs, astrocytes, oligodendrocytes, neuroblasts and neurons. For pancreatic organoid cells we used 20 cycles. It is advisable to test different cycle numbers between 18 and 25 before proceeding with the full protocol. A concentration of more than 0.3 ng/μL and the lack of fragments smaller than 500 bp are characteristics of a good cDNA profile.
    **Pause Point:** Store at 4°C up to 16 h or proceed directly with the purification of the cDNA.
    cDNA purification: Bead clean-up.
15. Equilibrate AMPure XP beads at 18°C–25°C for 30 min. Add 3 μL of beads to the mRNA plate (reaching 0.6× ratio of beads to sample volume).
16. Vortex for 30 s and spin down. Incubate 3 min. Repeat twice more to reach a total of 10 min of incubation at RT. Centrifuge the plate at 1,000 × *g* for 1 min.
  a. During the incubation time prepare 18 mL of EtOH 80% and aliquot 41 μL into a new 384-well plate.
17. Incubate the mRNA plate 2 min on magnet. Discard the SN and perform the following step twice (EtOH wash):
  a. Add 20 μL of EtOH 80% to the mRNA plate (on magnet). Wait 30 s and discard SN. Let the beads from the mRNA plate dry for 2 min, until no EtOH 80% is seen on the wells.
18. Remove mRNA plate from magnet. Add 8 μL of nuclease-free water. Vortex 30 s, spin down and incubate 1 min at RT. Repeat the vortexing and incubation two times more, to reach a total of 5 min incubation.
19. Place the plate on magnet for 2 min and transfer SN to a new plate (cDNA plate).
20. Perform quality control:
  a. Measure the concentration of 10 random samples (wells) with Qubit dsDNA HS Assay Kit as described by the manufacturer’s instructions (https://www.thermofisher.com/document-connect/document-connect.html?url=https://assets.thermofisher.com/TFS-Assets%2FLSG%2Fmanuals%2FQubit_dsDNA_HS_Assay_UG.pdf):
    i. Mix 1 μL of sample + 199 μL of a mix consisting of 199 μL of Qubit HS buffer + 1 μL of Qubit reagent. Vortex and measure on Qubit Fluorometer (Thermo Fisher Scientific).
  b. Load 1 μL of the 10 samples onto a Bioanalyzer High Sensitivity DNA chip (or TapeStation, Agilent) (concentration should not exceed 5 ng/μL, otherwise dilute in nuclease-free water before loading). Follow the manufacturer’s instructions (https://www.agilent.com/cs/library/usermanuals/public/G2938-90322_HighSensitivityDNAKit_QSG.pdf).
  c. Check the quality of the cDNA (see Figure 1 that displays good quality cDNA profiles, and troubleshooting 1).

**Figure 1:**
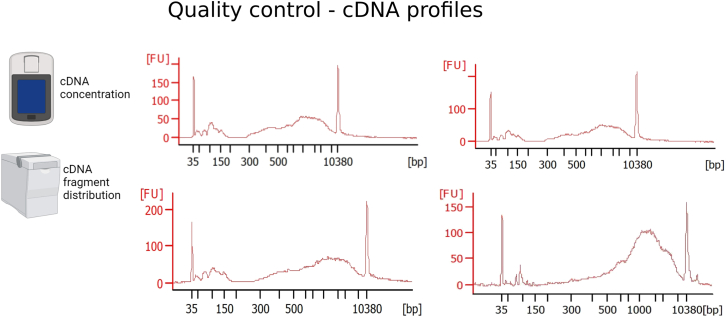
Expected outcomes after cDNA amplification Measure the concentration of the cDNA with a Qubit Fluorometer (Thermo Fisher Scientific) and the fragment distribution with Bioanalyzer or Tape Station (Agilent). cDNA profile of 4 random samples. Note that the proportion of primer dimer (50–150 bp) is lower than the one of the cDNA fragments (400–10000 bp). The proportion of primer dimers should not exceed the one showed here. Parts of this figure are created with BioRender.com.

***Optional:*** To get a better overview of the quality of the whole plate, the concentration of all the samples (384) can be measured using a fluorescent microplate reader (Biotek Synergy LX, Agilent) together with the Quant-IT PicoGreen dsDNA Assay-Kit. This also allows an optimal normalization of the cDNA before proceeding to the tagmentation.

For more information follow the manufacturer’s instructions (https://www.thermofisher.com/document-connect/document-connect.html?url=https://assets.thermofisher.com/TFS-Assets%2FLSG%2Fmanuals%2Fmp07581.pdf).

### Tagmentation-based library preparation

**Timing: 2 h**

Through these steps, the cDNA will be tagmented with sequencing adaptors and indexed. This allows the final multiplexing of the 384 or 768 single cells, depending on whether you plan to sequence 1 or 2 plates on the same transcriptomics sequencing run. For more information about the amount of input and cycle number required for the tagmentation process, please refer to the Smart-seq3 protocol.^5^^,^^6^

Tagmentation.

21. Normalize the cDNA to a concentration of 1 ng/μL. If the overall concentration of the plate is lower, normalize to 0.7 ng/μL and adjust appropriately the reaction volumes.

***Note:*** If a microplate reader is not available, average the concentration measurements from 10–15 random cells and then dilute the cDNA to the final concentration of 1 ng/μL with nuclease-free water. If a microplate reader is available, determine the cDNA concentrations and add the proper volumes of water per well in order to normalize the concentration of each well to 0.4–2.5 ng/μL. This can be done with Mantis.

22. Prepare the tagmentation master mix and aliquot 1.2 μL on a new plate (tagmentation plate).

**Table:** Tagmentation master mix

Reagent	Amount for 1 reaction (1×)	Amount for 1 plate (384 + 15%, i.e., 441.6×)
Tagment DNA buffer	0.8 μL	353.28 μL
Amplicon Tagment mix	0.4 μL	176.64 μL
Total	1.2 μL	529.92 μL

23. Add 0.4 μL of normalized cDNA (100–1000 pg of input) to the tagmentation plate. Vortex 30 s, and spin down. Repeat vortex and spin two times more. Incubate on thermocycler.

***Note:*** Input amounts of cDNA can vary. For a specific table describing the input amount for tagmentation please refer to the Smart-seq2^19^ and Smart-seq3^5^^,^^6^ papers. In addition, if having remaining TSO dimers from the cDNA amplification part, increase the input amount to compensate for their presence. The same procedure can be applied in case of obtaining suboptimal profiles after tagmentation (see troubleshooting).

**Table:** cDNA tagmentation. PCR cycling conditions

Steps	Temperature	Time	Cycles
Tagmentation	55°C	10 min	1
Hold	4°C

24. Add 0.4 μL of 0.2% SDS to the tagmentation plate. Vortex for 30 s and spin down. Incubate 5 min at 18°C–25°C.

***Optional:*** Instead of 0.2% SDS, NT buffer (Nextera XT DNA sample preparation kit) can be used.

**Table:** SDS 0.2%

Reagent	Amount for 1 reaction (1×)	Amount for 1 plate (384 + 15%, i.e., 441.6×)
SDS 0.2%	0.4 μL	70.66 μL

Library amplification.

25. Add 1.2 μL of Nextera PCR master mix (NPM) to the tagmentation plate.

***Optional:*** Alternatively, KAPA HiFi ready mix (2×) can be used instead of the Nextera PCR master mix. In this case use 3.2 μL instead of 1.2 μL for the final mix. Our experience shows that the results are similar by comparing the use of the 2 polymerases. If using the KAPA mix, make sure to keep the same proportion of beads to the sample volume in the further clean-up steps.

**Table:** Nextera PCR Master mix

Reagent	Amount for 1 reaction (1×)	Amount for 1 plate (384 + 15%, i.e., 441.6×)
Nextera PCR Mastermix	1.2 μL	529.92 μL

26. Add 0.8 μL of Nextera Primer index mix (i5 + i7 indexes combined at a concentration of 12.5 μM, see enclosed table with the index sequences: Table S1: mRNA index primers). Vortex 30 s, spin down and incubate on thermocycler.

**Figure 2:**
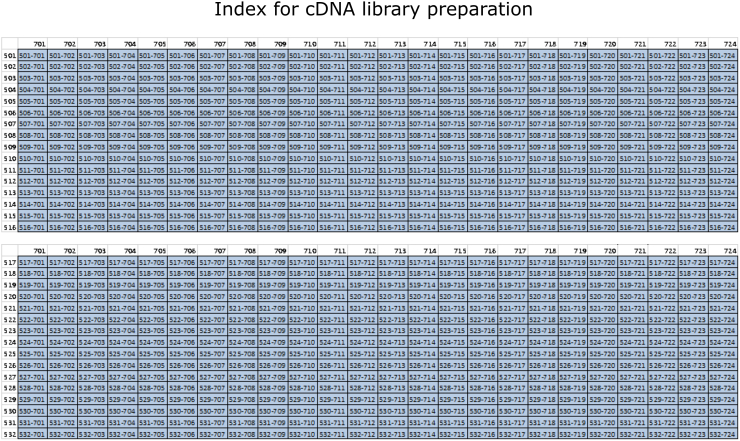
mRNA library amplification indexes The figure shows the possible combinations of i5 and i7 indexes in order to multiplex 768 cells on a single sequencing run.

***Note:*** Example layout of indexes for multiplexing 768 cells for the same sequencing run (Figure 2). This strategy uses combinatorial indexing and the numbers represent the indexes used. We provide the files with the index sequences in a format that can be easily used for ordering the oligos (5er and 7er indexes). The original design of the primers can be found here.^10^

**Table:** Library amplification. PCR cycling conditions with NPM

Steps	Temperature	Time	Cycles
Initial Extension	72°C	3 min	1
Initial Denaturation	98°C	3 min	1
Denaturation	98°C	10 s	6–16
Annealing	55°C	30 s	
Extension	72°C	30 s	
Final extension	72°C	5 min	1
Hold	4°C

**CRITICAL:** Cycle number can be adjusted according to the input of cDNA. For primary NSCs, astrocytes, oligodendrocytes, neuroblasts and neurons we have used 13 cycles for an input of 500 pg. For pancreatic organoids we have used 12 cycles for an input of 400 pg. However, the number of cycles and the amount of input depend on the cell type. When using KAPA instead of Nextera PCR Mastermix, the PCR conditions are different for certain steps, as follows. Denaturation: 98°C for 20, Annealing: 63°C for 15 s. For more information on input and cycle number please refer to the Smart-seq2 and Smart-seq3 protocols.^5^^,^^6^ Below is the table showing the cycle layout.

**Table:** Library amplification. PCR cycling conditions with KAPA

Steps	Temperature	Time	Cycles
Initial Extension	72°C	3 min	1
Initial Denaturation	98°C	30 s	1
Denaturation	98°C	20 s	6–16
Annealing	63°C	15 s	
Extension	72°C	30 s	
Final extension	72°C	3 min	1
Hold	4°C		

### cDNA library purification, quality control, and sequencing

**Timing: 4 h**

Through these steps the cDNA library will be purified and checked for quality control.

Purification by bead clean-up.

27. Equilibrate AMPure XP beads at RT for 30 min. Add 3 μL of beads to the tagmentation plate, reaching a 0.75× proportion.
28. Vortex for 30 s and spin down. Incubate 3 min.
  a. Repeat twice more to reach a total of 10 min of incubation at RT.
  b. Centrifuge the plate at 1,000 × *g* for 1 min.
  c. During the incubation time prepare 18 mL of EtOH 80% and aliquot 41 μL into a new 384-well plate.
29. Incubate the tagmentation plate 2 min on Magnet. Discard the SN perform the following step twice (EtOH wash):
  a. Add 20 μL of EtOH 80% to the tagmentation plate.
  b. Wait 30 s and discard SN.

***Note:*** Let the beads from the tagmentation plate dry for 2 min, until no EtOH 80% is seen on the wells.

30. Remove the tagmentation plate from the magnet.
  a. Add 7 μL of nuclease-free water.
  b. Vortex 30 s, spin down and incubate 1 min.
  c. Repeat the vortexing and incubation two times more, to reach a total of 5 min incubation.
31. Place plate on magnet for 2 min and transfer SN to a new plate (purified mRNA library plate).
32. Repeat the purification by adding 4.5 μL of AMPure beads (0.65×). Elute in 7 μL of nuclease-free water.
33. Perform Quality control similarly to the quality control of the cDNA (step 20).

Pooling of final library.

34. Normalize tagmented cDNA to a concentration of 0.5 ng/μL and pool 5 μL of every well into a final tube (for concentration measurement and normalization please use a similar workflow as step 21). Pooling procedure:
  a. Use Mosquito HV (SPT Labtech) to aspirate 5 μL from every column of the source plate (sample plate) and transfer to one column of a deep-well plate (called pool plate). Always change tips between transfers, to avoid contamination between the samples.
  b. After pooling into a single column, use a normal 1000 μL pipette to transfer the contents into a 2 mL microcentrifuge tube. The ideal amount should be 384∗5=1920 μL. Mix the solution by pipetting up and down 10 times. The volume might be sometimes less than 1920 μL.
  c. Transfer 960 μL into a new 2 μL microcentrifuge tube. Now the pool should be divided on 2 tubes.
35. Perform a 1× bead cleanup.
  a. Measure exactly the volume of pool in every tube and add the same amount of AMPure XP beads in order to reach a 1× proportion. Mix by pipetting.
  b. Incubate 10 min at RT. Place the tube 3 min on magnet and discard SN.
  c. Wash two times with EtOH 80% and resuspend in 35 μL of nuclease-free water.

**CRITICAL:** When adding the water, resuspend first the beads with water on one tube, and then transfer the resuspended beads to the second tube (which has the dry beads). With this procedure, the two tubes are now fused into one, and the pool is resuspended in 35 μL of water containing the pooled cDNA library.

36. Measure the DNA concentration of the final pool using Qubit dsDNA HS Assay Kit as described before. Analyze the fragment distribution with Bioanalyzer or TapeStation (Agilent).

#### Sequencing

37. The pool can be sequenced on both patterned (NextSeq 2000 (https://emea.support.illumina.com/sequencing/sequencing_instruments/nextseq-1000-2000/documentation.html) and NovaSeq 6000 (https://emea.support.illumina.com/sequencing/sequencing_instruments/novaseq-6000/documentation.html)) and non-patterned flow cell sequencers (NextSeq550 (https://emea.support.illumina.com/sequencing/sequencing_instruments/nextseq-550/documentation.html) and HiSeq2000 (https://emea.support.illumina.com/sequencing/sequencing_instruments/hiseq_2000/documentation.html)).

***Note:*** For a 1-plate run (384 cells) it is advisable to sequence either on a NextSeq2000 P1 100 cycles (100 million reads) or on a NexSeq 550 75 bp Paired End Mid output (MO) (104 million reads), in order to reach 300,000 reads per cell. For a 2-plate run (786 cells) the NS550 75 bp Paired End High output (HO) (320 Million reads) or the NS2000 P2 100 cycles (400 million reads) are recommended. Patterned-flow cell sequencers offer an improved sequencing yield together with a much lower cost per sequenced read. We think this improvement will allow users to reduce the price of their sequencing runs. It is always recommended to spike in PhiX to the sample before sequencing. PhiX is an adapter-ligated library used as a control for Illumina sequencing runs. It offers benefits for sequencing and alignment. We have used 1% as a final concentration of PhiX in our sequencing runs for the NS550. When sequencing on a NS2000, users should add PhiX at 8%–10% final concentration, instead of the 1% that is used for non-patterned flow cells. The combinatorial indexing strategy for mRNA sequencing remains the same as in the original protocol.

### Genomic part

#### gDNA purification and bisulfite conversion

**Timing: 4 h**

Through these steps the gDNA will be purified and bisulfite-converted. In this process, the gDNA will be fragmented and non-methylated cytosines will be chemically modified into uracils. In the conversion step, unmethylated cytosines will be deaminated. In order to save time, this step can be performed on the same day as the final part of the cDNA library preparation.

gDNA purification.

38. Equilibrate AMPure XP beads at RT for 30 min.
  a. Add 15 μL of beads to the gDNA plate (reaching 0.68× proportion) which was frozen and stored at −20°C on step 11.
39. Vortex for 30 s and spin down.
  a. Incubate 10 min at RT.
  b. Repeat twice more to reach a total of 30 min of incubation.
  c. Centrifuge the plate at 1,000 × *g* for 1 min. During the incubation time, prepare 18 mL of EtOH 80% and aliquot 41 μL into a new 384-well plate.
40. Incubate 2 min on magnet. Discard SN and perform the following step twice (EtOH wash):
  a. Add 20 μL of EtOH 80% to the gDNA plate. Wait 30 s and transfer SN to waste plate. Let the beads dry for 2 min, until no EtOH 80% is seen on the wells.
41. Remove gDNA plate from magnet.
  a. Add 2.5 μL of nuclease-free water.
  b. Vortex 30 s, spin down and incubate 1 min.
  c. Repeat the vortexing and incubation twice more, to reach a total of 5 min incubation.

**CRITICAL:** Do not aspirate the water from the gDNA plate.

Bisulfite conversion.

42. Place gDNA plate on magnet. Add 16 μL of pre-warmed (37°C) CT conversion reagent. Do not vortex (in order to avoid the beads mixing with the conversion reagent). Incubate on thermocycler as follows:

**Table:** Bisulfite Conversion. PCR cycling conditions

Steps	Temperature	Time	Cycles
Initial Denaturation	98°C	8 min	1
Conversion	64°C	180 min	1
Hold	4°C

**Pause Point:** gDNA plate can be left 16–24 h at 4°. Do not exceed 24 h of incubation at 4°C.

#### Desulfonation, 1° strand synthesis, and exonuclease treatment

**Timing: 8 h**

Through these steps the converted gDNA will be purified and the desulfonation process will take place, whereby the sulfite moiety from the cytosines will be removed to generate uracil bases. At the end the process of DNA amplification will start, with the 1° strand synthesis and the remaining free primers will be excised by exonuclease treatment.

gDNA clean-up with MagBinding beads.

43. Prepare Zymo MagBinding bead mix by mixing beads with M-Binding buffer in the following proportion:

**Table:** MagBinding bead mix

Reagent	Amount for 1 reaction (1×)	Amount for 1 plate (384 + 15%, X441.6)
MagBinding beads	5 μL	2208 μL
M-Binding buffer	70 μL	30912 μL
Total	75 μL	33120 μL

44. Aliquot 75 μL of bead mix into a 384-deep well plate. Also prepare an EtOH plate with 41 μL per well.
45. Perform the binding of the Bead mix with the gDNA.
  a. Transfer 25 μL of bead mix from the deep-well plate to the gDNA plate, perform a pipette mix and transfer 45 μL back to the deep-well plate.
  b. Add again 25 μL of bead mix to the gDNA plate, mix and transfer 25 μL back to the deep-well plate.
  c. Rinse the gDNA plate with 20 μL of bead mix and transfer all the remaining liquid to the deep-well plate. Vortex the deep-well plate 30 s and incubate 5 min at 18°C–25°C.
46. Procedure for discarding of SN and washing of the gDNA:
  a. Transfer 33.3 μL of bead mix from the deep-well plate to the gDNA plate. Spin down the gDNA plate at 1,000 × *g* for 1 min and then put plate on the magnet. Incubate 2 min. Discard SN.
  b. Repeat step a twice more and make sure that there are no beads remaining on the deep-well plate.
  c. Perform the following step **once** (EtOH wash):
    i. Add 20 μL of EtOH 80% to the gDNA plate. Wait 30 s and transfer SN to waste plate. Let the beads air dry at 18°C–25°C for 2 min.

Desulfonation and 1° strand synthesis.

47. Add 25 μL of M-Desulfonation to the gDNA plate. Vortex 30 s and spin the samples shortly. Incubate 15 min at 18°C–25°C. Meanwhile, prepare the 1° strand synthesis mix:

**Table:** 1° strand synthesis master mix

Reagent	Initial concentration	Amount for 1 reaction (1×)	Amount for 1 plate (384 + 15%, i.e., 441.6×)	Final concentration
ddH_2_O		7.9 μL	3488.64 μL	
Blue buffer (Part of the Klenow enzyme kit, see key resources table)	10 ×	1 μL	441.6 μL	1×
dNTP mix	10 mM	0.4 μL	176.64 μL	0.4 mM
Preamplification Oligo (1° Strand oligo)	10 μM	0.4 μL	176.64 μL	0.4 μM
Total		9.7 μL	4283.52 μL

48. Centrifuge the gDNA plate at 1,000 × *g* for 1 min and place on magnet for 2 min. Perform two EtOH washes (as explained on step 46.c.I).
49. To dry the beads, put the gDNA plate **without foil** on a thermocycler with the lid open for 8 min at 55°C.
50. Add 9.7 μL of First Strand synthesis mix and seal the plate. Vortex 30 s, spin down briefly and incubate 1 min. Repeat the vortexing step twice more to reach a total of 5 min incubation.
51. Incubate the sealed plate 5 min at 55°C on a thermocycler with the lid closed.
  a. Centrifuge plate at 1,000 × *g* for 1 min.
  b. Put on magnet for 2 min and transfer SN to a new, fresh plate.
  c. Incubate 3 min at 65°C on a thermocycler. Immediately cool on ice.
52. Add 0.3 μL of Klenow (50 U/μL). Vortex 10 s and spin down. Incubate on thermocycler as follows:

**Table:** 1° strand synthesis – short PCR. PCR cycling conditions - 1 part

Steps	Temperature	Time	Cycles
Initial Incubation	4°C	5 min	1
Slow ramping	4°C–37°C at a rate of 4°C/min (0.06°C/s)	8.25 min	1
Extension	37°C	30 min	1
Hold	4°C

53. Incubate at 95°C for 45 s and afterward cool to 4°C on an ice block.
54. Add 0.6 μL of “first strand extra cycles” mix.

**Table:** 1° strand synthesis “extra cycles” master mix

Reagent	Initial concentration	Amount for 1 reaction (1×)	Amount for 1 plate (384 + 15%, i.e., 441.6×)
ddH_2_O		0.156 μL	68.89 μL
Blue Buffer	10×	0.06 μL	26.50 μL
dNTP mix	10 mM	0.024 μL	10.60 μL
Preamplification Oligo (1° Strand oligo)	10 μM	0.24 μL	105.98 μL
Klenow exo-	50 U/μL	0.12 μL	52.99 μL
Total		0.6 μL	264.96 μL

55. Vortex 10 s, spin down and incubate as follows:

**Table:** 1° Strand synthesis – short PCR. PCR cycling conditions - 2 Part

Steps	Temperature	Time	Cycles
Initial incubation	4°C	5 min	1
Slow ramping	4°C–37°C at a rate of 4°C/min (0.06°C/s)	8.25 min	1
Extension	37°C	30 min	1
Hold	4°C

56. Go to step “53” and repeat steps 53–55 three more times. For the last repetition, use the following program for the thermocycler:

**Table:** 1° strand synthesis – long PCR. PCR cycling conditions

Steps	Temperature	Time	Cycles
Initial Incubation	4°C	5 min	1
Slow ramping	4°C–37°C at a rate of 4°C/min (0.06°C/s)	8.25 min	1
Extension	37°C	90 min	1
Hold	4°C

57. In total you have done 5 PCRs.

**Pause Point:** You can incubate the plate at 4°C between 16–24 h or proceed to the next step.

58. Add 15 μL of exonuclease mix. Vortex 30 s and spin down. Incubate on thermocycler at 37°C for 60 min with the lid at 50°C. The total volume in plate is now 27.4 μL.

**Pause Point:** The plate can be stored up to 1 month at −20°C.

**Table:** Exonuclease mix

Reagent	Initial concentration	Amount for 1 reaction (1×)	Amount for 1 plate (384 + 15%, i.e., 441.6×)
ddH_2_O		14.5 μL	6403.2 μL
Exonuclease I	20 U/μL	0.5 μL	220.8 μL
Total		15 μL	6624 μL

#### 1° strand product purification, 2° strand synthesis and purification, and library amplification

**Timing: 4 h**

Through these steps the 1° strand product will be purified and further amplified using a second primer (Adapter 2 oligo). This product will be purified at the end. On the final step, the library will be amplified by using indexed primers (Unique dual index, UDI) and the KAPA enzyme.

1° strand purification.

59. Equilibrate AMPure XP beads at RT for 30 min. Add 17 μL of beads to the gDNA plate (reaching 0.62× bead proportion).
60. Vortex for 30 s and spin down. Incubate 3 min. Repeat twice more to reach a total of 10 min of incubation. Centrifuge the plate at 1,000 × *g* for 1 min.
  a. During the incubation time prepare 18 mL of EtOH 80% and aliquot 41 μL into a new 384-well plate.
  b. During the incubation time also prepare the 2° strand synthesis master mix.

**Table:** 2° strand synthesis master mix

Reagent	Initial concentration	Amount for 1 reaction (1×)	Amount for 1 plate (384 + 15%, i.e., 441.6×)	Final concentration
ddH_2_O		9.8 μL	4327.68 μL	
Blue Buffer	10 ×	1.22 μL	538.75 μL	1 ×
dNTP mix	10 mM	0.49 μL	216.38 μL	0.4 mM
Adaptor 2 Oligo (2° Strand oligo)	10 μM	0.49 μL	216.38 μL	0.4 μM
Total		12 μL	5299.2 μL

61. Incubate 2 min on Magnet. Transfer the SN to the waste plate and perform the following step twice (EtOH wash).
  a. Add 20 μL of EtOH 80% to the mRNA plate. Wait 30 s and transfer SN to waste plate. Let the beads from the gDNA plate dry for 2 min, until no EtOH 80% is seen on the wells.

2° strand amplification.

62. Remove gDNA plate from magnet. Add 12 μL of 2° strand synthesis master mix. Vortex 30 s, spin down and incubate 1 min. Repeat the vortexing and incubation two times more, to reach a total of 5 min incubation. Transfer the content of every well to a new plate (without magnet).
63. Incubate at 95°C for 45 s and afterward cool to 4°C on an ice block.
64. Add 0.3 μL of Klenow-exo. Vortex 10 s and spin down. The total volume is 12.3 μL. Incubate on thermocycler as follows:

**Table:** 2° strand synthesis – long PCR. PCR cycling conditions.

Steps	Temperature	Time	Cycles
Initial Incubation	4°C	5 min	1
Slow ramping	4°C–37°C at a rate of 4°C/min (0.06°C/s)	8.25 min	1
Extension	37°C	90 min	1
Hold	4°C		

2° strand purification.

65. Equilibrate PEG buffer 18% at RT for 30 min. Add 12 μL of nuclease-free water and mix. Add 18 μL of PEG buffer and mix again (the bead proportion is now 0.74×).
66. Vortex for 30 s and spin down. Incubate 3 min. Repeat twice more to reach a total of 10 min of incubation. Centrifuge the plate at 1,000 × *g* for 1 min.
  a. During the incubation time prepare 18 mL of EtOH 80% and aliquot 41 μL into a new 384-well plate.
  b. During the incubation time also prepare the library amplification master mix (KAPA mix).

**Table:** Library amplification master mix

Reagent	Initial concentration	Amount for 1 reaction (1×)	Amount for 1 plate (384 + 15%, i.e., 441.6×)	Final concentration
ddH_2_O		5.75 μL	2539.2 μL	
KAPA HiFi ready mix	2×	6.25 μL	2760 μL	1×
Total		12 μL	5299.2 μL

67. Incubate 2 min on magnet. Transfer the SN to the waste plate and perform the following step twice (EtOH wash).
  a. Add 20 μL of EtOH 80% to the mRNA plate. Wait 30 s and transfer SN to waste plate. Let the beads from the gDNA plate dry for 2 min, until no EtOH 80% is seen on the wells.

#### Library amplification and purification

68. Add 12 μL of KAPA mix, vortex 30 s, and spin down. Incubate 10 min at RT to elute the DNA.
69. Add 0.5 μL of Index mix (combinatorial indexing of the primer mix for gDNA, see key resources table, Figure S1 and enclosed file; Table S2: gDNA index primers).

***Note:*** This allows the full multiplexing of 384 cells on the gDNA level, with no reduction in sequencing quality and decrease in sequencing costs. Vortex 30 s and spin down. Incubate on thermocycler as follows.

**Table:** gDNA library amplification. PCR cycling conditions

Steps	Temperature	Time	Cycles
Initial denaturation	95°C	2 min	1
Denaturation	94°C	80 s	14
Annealing	65°C	30 s	
Extension	72°C	30 s	
Final extension	72°C	3 min	1
Hold	4°C

70. Equilibrate PEG buffer 18% at RT for 30 min. Add 12 μL of nuclease-free water and mix. Add 18 μL of PEG and mix again (the bead proportion is now 0.73×).
71. Vortex for 30 s and spin down. Incubate 3 min. Repeat twice more to reach a total of 10 min of incubation. Centrifuge the plate at 1,000 × *g* for 1 min.
72. Incubate 2 min on magnet. Transfer the SN to the waste plate and perform the following step twice (EtOH wash).
  a. Add 20 μL of EtOH 80% to the mRNA plate. Wait 30 s and transfer SN to waste plate. Let the beads from the gDNA plate dry for 2 min, until no EtOH 80% is seen on the wells.
73. Add 7 μL of nuclease-free water. Vortex 30 s, spin down and incubate 1 min. Repeat the vortexing and incubation twice more, to reach a total of 5 min incubation.
74. Place plate on magnet for 2 min and transfer SN to a new plate (final gDNA plate).
75. Perform quality control similarly to the quality control of the cDNA in step 20.

Pooling of final gDNA library.

76. Normalize the gDNA library to a concentration of 0.5 ng/μL and pool 3 μL of every well into a final tube (for concentration measurement and normalization, please use a similar workflow as in step 21; for pooling, use a similar workflow as in step 34).
77. Perform a 1× bead clean-up as described for the tagmented cDNA in step 27.
78. Measure the DNA concentration of the final pool with Qubit dsDNA HS Assay Kit as described before. Analyze the fragment distribution with Bioanalyzer.

#### Sequencing

79. The pool can be sequenced on an Illumina NextSeq 1000–2000 sequencer (or any other patterned-flow cell sequencer: HiSeq X, HiSeq 3000/4000, NovaSeq 6000). For 96 cells per sequencing run, it is advisable to aim for 4–6 million reads per cell, in order to reach the minimum of 50.000 CpGs per cell. It should always be sequenced paired-end and with a read length of at least 75 bp. Note that the gDNA libraries can also be sequenced on non-patterned flow cell sequencers (NextSeq550), since the samples have UDI. However, we prefer to use patterned-flow cell sequencer, since the output of sequencing data is more cost-effective. We have used 15% as a final concentration of PhiX in our sequencing runs for the gDNA part. Note that this high concentration is due to the low diversity of bisulfite treated gDNA libraries.

### Alignment and quantification of transcriptomic data

**Timing: 4 h**

Through these steps the transcriptomic data will be mapped to the reference genome. This allows the quantification of the gene expression profile of single cells.

Follow the steps recommended by^5^^,^^6^ in the SmartSeq3 protocol. For convenience, we briefly recapitulate these methods.

80. Convert Binary Base Call (BCL) files to FASTQ format with the bcl2fastq software provided by Illumina.
  bcl2fastq --use-bases-mask Y150N,I8,I8,Y150N --no-lane-splitting --create-fastq-for-index-reads -R /run_directory/
  where /run_directory/ is the directory containing the BCL files.
  a. Inspect the quality of several FASTQ files with FASTQC (https://www.bioinformatics.babraham.ac.uk/projects/fastqc/).
  b. All FASTQ files may then be demultiplexed, mapped, and quantified with the software zUMIs (https://github.com/sdparekh/zUMIs). To configure your zUMIs run, you will need to prepare a YAML file that denotes all parameters and paths.^5^^,^^6^ provide the following template for a typical Smart-seq3 run:
    project: Smartseq3
    sequence_files:
     file1:
      name: /smartseq3/fastq/Undetermined_S0_R1_001.fastq.gz
      base_definition:
       - cDNA(23-150)
       - UMI(12-19)
      find_pattern: ATTGCGCAATG
     file2:
      name: /smartseq3/fastq/Undetermined_S0_R2_001.fastq.gz
      base_definition:
       - cDNA(1-150)
     file3:
      name: /smartseq3/fastq/Undetermined_S0_I1_001.fastq.gz
      base_definition:
       - BC(1-8)
     file4:
      name: /smartseq3/fastq/Undetermined_S0_I2_001.fastq.gz
      base_definition:
       - BC(1-8)
     reference:
      STAR_index: /resources/genomes/Mouse/STAR5idx_noGTF/
      GTF_file: /resources/genomes/Mouse/Mus_musculus.GRCm38.91.gtf
      additional_STAR_params: '--clip3pAdapterSeq CTGTCTCTTATACACATCT'
      additional_files:
       - /resources/genomes/spikes/ERCC92.fa
     out_dir: /smartseq3/zUMIs/
     num_threads: 20
     mem_limit: 50
     filter_cutoffs:
      BC_filter:
       num_bases: 3
       phred: 20
     UMI_filter:
       num_bases: 2
       phred: 20
     barcodes:
      barcode_num: ∼
      barcode_file: /smartseq3/expected_barcodes.txt
      automatic: no
      BarcodeBinning: 1
      nReadsperCell: 100
      demultiplex: no
     counting_opts:
      introns: yes
      downsampling: '0'
      strand: 0
      Ham_Dist: 1
      write_ham: no
      velocyto: no
      primaryHit: yes
      twoPass: no
     make_stats: yes
     which_Stage: Filtering
     samtools_exec: samtools
     pigz_exec: pigz
     STAR_exec: STAR
     Rscript_exec: Rscript
81. After substituting the appropriate paths in this template, your zUMIs run may be started:

zUMIs.sh –c -y Smartseq3_config.yaml

### Alignment and quantification of epigenomic data

**Timing: 3 days**

Through these steps the epigenomic data will be mapped to the reference genome. This allows the quantification of the methylation and chromatin accessibility profile of single cells.

The total time will depend on the power of the computer cluster used for processing the data. On average we find that 3 days are enough to map 96 cells. This part of the protocol is based on recommendations described in the original scNMT-seq paper.^4^ We recommend Bismark (https://www.bioinformatics.babraham.ac.uk/projects/bismark/) (10.1093/bioinformatics/btr167) for mapping and quantification of genomic reads, since Bismark can quantify both CpG-methylation and GpC-methylation. Since mapping of bisulfite-converted whole-genome reads is computationally expensive, you should consider performing this analysis on a large compute cluster or a similar system. The following steps are required for every cell, so it’s advisable to use a workflow manager like Snakemake or Nextflow to process multiple cells in parallel. It is not advisable to process FASTQ-files with extremely low read number (e.g., below 10.000 reads) since these cells will not pass later quality checks anyways, and they might cause crashes in later processing steps.

82. Follow the steps recommended by Illumina to demultiplex your sequencing data and to convert them to FASTQ format.
83. Prepare the genome for methylation-aware alignment with Bismark. This step is only required once.

bismark_genome_preparation --path_to_aligner /usr/bin/bowtie2/ --verbose /genomes/homo_sapiens/GRCh37/

84. Trim sequencing adapters with trim_galore (https://www.bioinformatics.babraham.ac.uk/projects/trim_galore/) as follows:

trim_galore --output_dir trimmed/ --paired cell1_read1.fastq.gz cell1_read2.fastq.gz

85. Map sequencing reads to the genome in single-end non-directional mode:

bismark --non_directional --genome /genomes/homo_sapiens/GRCh37/ cell1_read1.fastq.gz --output_dir mapped/

bismark --non_directional --genome /genomes/homo_sapiens/GRCh37/ cell1_read2.fastq.gz --output_dir mapped/

86. Remove PCR duplicates:

deduplicate_bismark mapped/cell1_read1_bismark_bt2.bam --single --bam --output_dir mapped/ --outfile read1_deduplicated.bam

deduplicate_bismark mapped/cell1_read2_bismark_bt2.bam --single --bam --output_dir mapped/ --outfile read2_deduplicated.bam

87. Merge mates:

samtools merge merged/cell1_merged.bam cell1_read1_deduplicated.bam cell1_read2_deduplicated.bam

88. Quantify DNA methylation at all cytosines.

bismark_methylation_extractor --gzip --CX --output methylation-calls/ merged/cell1_merged.bam

89. Use Bismark’s NOMe-seq option to distinguish between CpG and GpC methylation:

coverage2cytosine --nome-seq --dir methylation-calls/ --genome_folder /genomes/homo_sapiens/GRCh37/ --output cell1 methylation-calls/cell1_merged.bismark.cov.gz

## Expected outcomes

### cDNA amplification

**cDNA profile after amplification:** The profiles should look as indicated in Figure 1. The presence of TSO dimers at around 80–150 bp in high proportion might hinder the downstream processing of the sample, in particular the tagmentation process. In this case, perform the TSO-wash as described previously. In addition, to obtain a good profile, it is advisable to reduce the time that the beads are without liquid (in particular in the mRNA and gDNA separation steps) and to pre-aliquot reagents in fresh 384-well plates in order to increase the speed of reagent transfer (see troubleshooting for more information). New data generated with this protocol can be found here.^1^^,^^2^

### cDNA library preparation

**Figure 3:**
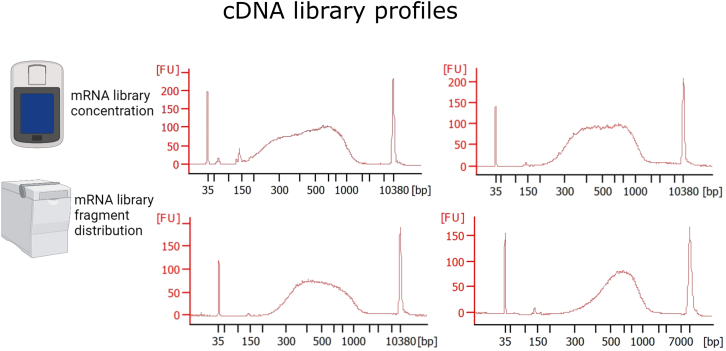
cDNA library profile of 4 random samples Measure the concentration with a Qubit Fluorometer (Thermo Fisher Scientific) and the fragment distribution with Bioanalyzer or Tape Station (Agilent). The average fragment size should vary between 400–900 bp. If primer dimers are still present, perform one 0.8× purification. Parts of the figure are created with BioRender.com.

**cDNA library profile:** The profiles should look as shown in Figure 3. An average fragment size of 500–900 bp is expected. If primer dimers are still present, perform another 0.8× purification.

### cDNA library pool

**Figure 4:**
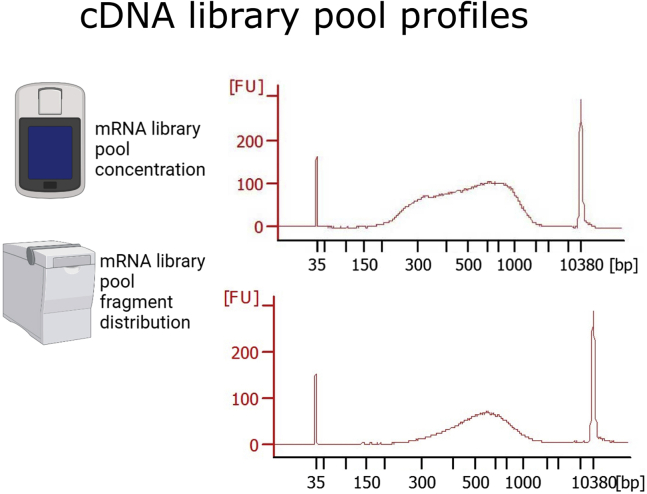
Pooled cDNA libraries Measure the concentration with a Qubit Fluorometer (Thermo Fisher Scientific) and the fragment distribution with Bioanalyzer or Tape Station (Agilent). Here two random mRNA library pools are shown. The average fragment size should be between 400–900 bp free of fragment smaller than 200 bp. Parts of the figure are created with BioRender.com.

**Pooled cDNA library profile:** The profile should look as shown in Figure 4. An average fragment size of 400–900 bp is expected. If primer dimers are still present, repeat again a 1× purification. On the final pool there should not be free adaptors (fragments of size less than 200 bp), as they will interfere with the sequencing.

### gDNA library

**Figure 5:**
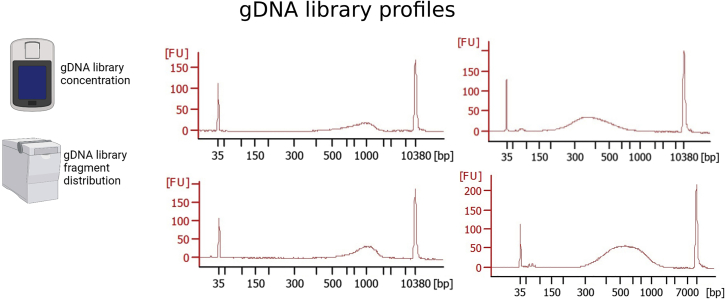
gDNA library profile of 3 random samples Measure the concentration of the gDNA with a Qubit Fluorometer (Thermo Fisher Scientific) and the fragment distribution with Bioanalyzer or Tape Station (Agilent). The average fragment size should vary between 500 and 900 bp. Parts of the figure are created with BioRender.com.

**gRNA library profile:** The profiles should look as shown in Figure 5. An average fragment size of 500–900 bp is expected. We had some runs with an average fragment size of 500 bp and some other runs with an average fragment size of 800–900 bp. For both neural cells and pancreatic organoid cells, we have used 14 cycles. Presence of fragment at ∼200–300 bp indicates the presence of primer concatemers, which might reduce the quality of the data.

### gDNA library pool

**Figure 6:**
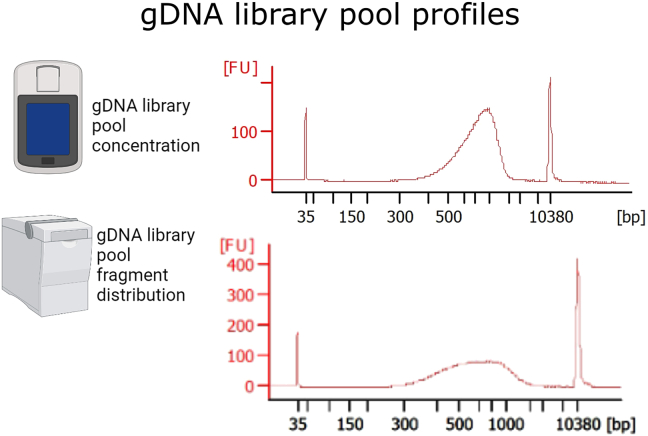
Pooled gDNA library profiles of two random samples Measure the concentration of the gDNA with a Qubit Fluorometer (Thermo Fisher Scientific) and the fragment distribution with Bioanalyzer or Tape Station (Agilent). The average fragment size should be between 500–900 bp. Parts of the figure are created with BioRender.com.

**gRNA library pool profile:** The profiles should look as shown in Figure 6. An average fragment size of 500–900 bp is normal. New gDNA data generated with this combinatorial indexing protocol can be found here.^1^^,^^2^ The combinatorial indexing data (384 cells per sequencing run) has a similar quality than the unique-dual indexed data (96 cells per run), since both types of experiments could be seamlessly integrated (for more information see supplementary table 2 of ref.^2^). An advantage of the combinatorial indexing approach is the reduced input requirement per well, as 384 wells are pooled for a single sequencing run instead of the standard 96. This increased multiplexing capacity reduces the need for extensive PCR amplification during library preparation.

### Alignment of transcriptomic data

**Figure 7:**
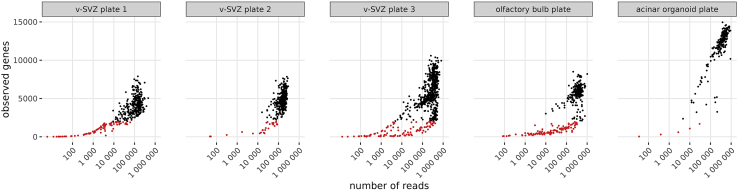
Quality metrics of single cell transcriptomes from five different plates Cells with less than 2000 observed genes are highlighted in red. These cells are candidates for quality filtering. Note that the in vitro acinar organoid cells appear to express more genes than murine in vivo cells since this difference cannot be explained by read number alone.

zUMIs will create a directory zUMIs_output/expression that contains the single-cell RNA sequencing (scRNA-seq) count matrix in .loom and .rds formats for standard scRNA-seq analysis in R or Python. As is common practice, we recommend to filter cells with an unusually low number of observed genes. Note that the gene number depends not just on RNA quality and sequencing depth, but also on the overall transcriptional activity of the sample (Figure 7).

### Alignment of epigenomic data

This workflow will produce two output files per cell, methylation-calls/cell1.NOMe.CpG.cov and methylation-calls/cell1.NOMe.GpC.cov, which list the methylation level at all CpG sites and GpC sites. Downstream analysis of these raw data may be performed with R or Python.

**Figure 8:**
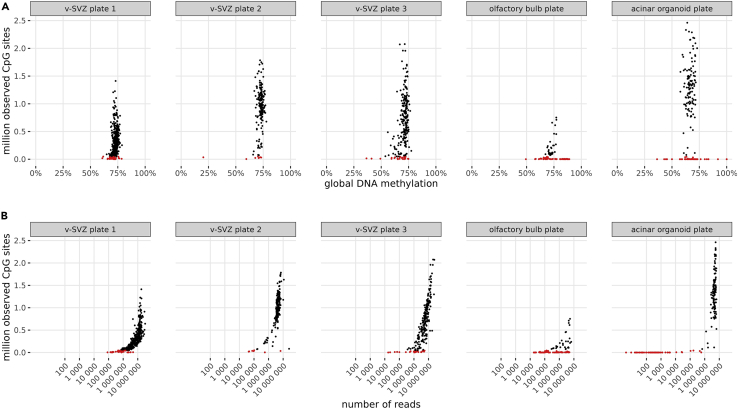
Quality metrics of single cell methylomes from five different plates (A) Observed CpG sites per cell versus global DNA methylation. Cells with less than 50,000 observed CpG sites are highlighted in red. These cells are candidates for quality filtering. (B) Observed CpG sites per cell versus number of reads. This shows that with at least 3–4 million reads per cell, the number of CpGs pass the lower threshold of 50,0000 CpGs.

For quality filtering, we recommend to discard cells with less than 50.000 observed CpG sites, and possibly cells with extremely high or low global DNA methylation. Depending on DNA quality, sequencing depth, and the proportion of PCR duplicate reads, the number of observed CpG sites may vary between experiments (Figure 8).

**Figure 9:**
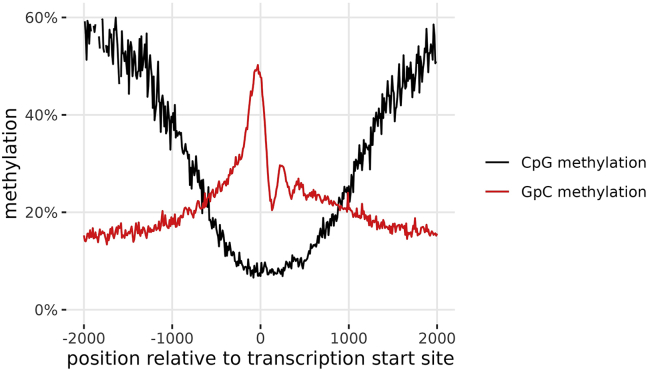
Average methylation of all transcription start sites one a single high-quality cell Genomic position is binned in bins of 10 bp width.

It may also be useful to inspect DNA methylation patterns and chromatin accessibility patterns at transcription start sites, to judge the quality of the data. In mammalian cells, the average transcription start site should be less methylated and more accessible than neighboring regions (Figure 9).

## Limitations

The updated version of the protocol allows the processing of 384 cell per protocol run, for mRNA as well as for gDNA. To increase the number of cells to be processed and sequence, the gDNA indexes can be multiplexed to reach 768 cells per sequencing run. The protocol is also laborious and might take to up to 1 week. To reduce time, the use of the liquid handlers is recommended. As explained before, the gDNA purification and conversion step can be done on the last day of the mRNA library part, in order to save one day of work.

## Troubleshooting

### Problem 1

High proportion of primer or TSO dimers after cDNA amplification (steps 13 and 14).

### Potential solution

• Perform TSO-wash (See step 13).
• Prealiquot the G&T wash buffer in a fresh plate. That enables an efficient separation of the gDNA and the mRNA and also reduces the time that the OligodT beads are exposed to air.
• Increase the number of PCR cycles in order to amplify more the cDNA.

### Problem 2

Primer dimer presence after cDNA tagmentation or suboptimal profiles (steps 21–33).

### Potential solution

• Perform another 0.6× bead cleanup in order to eliminate the presence of primer dimers.
• Increase the input of cDNA for tagmentation.

### Problem 3

Small average fragment size on gDNA library (200–300 bp, steps 68–75).

### Potential solution

• The initial material (gDNA) is in low concentration. Increase the incubation time with water from 30 min to 45 min on the gDNA purification step (step 39).
• Decrease the proportion of beads in the purification steps of the gDNA library to 0.6×.

### Problem 4

Low amount of gDNA library (less than 0.2 ng/μL, steps 68–75).

### Potential solution

Repeat library amplification with 2–4 cycles.

### Problem 5

High average fragment size on gDNA library (1200–1400 bp, steps 68–75).

### Potential solution

The conversion was not efficient. However, the library is still sequence-ready. For the next trial use freshly prepared conversion reagent and make sure that you don’t see crystals on the solution before aliquoting into a new plate.

### Problem 6

The 0.3 μL of klenow is too little to be dispensed and checked in Mantis (steps 52 and 64)

### Potential solution

After preparing the 1st and 2nd strand synthesis master mix, take out 322 μL (0,7 μL∗460) of master mix and transfer to a new tube. Then add 136 μL (0.3 μL∗460) Klenow to the 322 μL of the master mix epi. In this way, you would be able to deliver 1 μL of the Klenow + Master mix in the well, instead of 0.3 μL, making the workflow easier.

### Problem 7

There is still primer dimers at high concentration with respect to cDNA after the purification and quality control (step 20).

### Potential solution

Perform a second 0.6× purification, and elute in 6 μL of water.

## Resource availability

### Lead contact

Further information and requests for resources and reagents should be directed to and will be fulfilled by the lead contact, Ana Martin-Villalba (a.martin-villalba@dkfz-heidelberg.de).

### Technical contact

Request for resources and questions about the protocol can be fulfilled by the technical contact, Santiago Cerrizuela (s.cerrizuela@dkfz-heidelberg.de)

### Materials availability

This study did not generate new unique reagents.

### Data and code availability

All sequencing data are available at the NCBI Gene Expression Omnibus (GEO) under the SuperSeries accession GSE210806. The four brain data sets depicted in Figure 7 correspond to plates pD, pH, pI, and pE. The code used to process scNMT-seq data is provided as part of the protocol.

## Acknowledgments

We would like to thank the colleagues of the Molecular Neurobiology Division for fruitful discussions regarding the optimization of the protocol and Jan Philipp Mallm at the Single-Cell Open Lab at the DKFZ for providing the dual indexing primers used in the gDNA part. We thank S. Wolf & D. Helm from the DKFZ Genomics and Proteomics Core Facility; S. Schmitt from the DKFZ Flow Cytometry Core Facility, and the DKFZ Center for Preclinical Research. This work was supported by the 10.13039/100010663European Research Council (ERC; REBUILD_CNS).

## Author contributions

Conceptualization: A.M.-V., S.C., and O.K. Writing – review and editing: S.C., L.P.M.K., O.K., and A.M.-V. Methodology – performing the scNMT protocol with different cell types: S.C., T.E., A.S., J.S., and A.K. Development of the miniaturized protocol: S.C., O.K., and T.E. Design and implementation of troubleshooting alternatives: A.S.-M. and A.S. Establishment of pancreatic organoid culture: J.B. and A.S. Formal analysis – data processing and statistical analysis: L.P.M.K. Funding acquisition: A.M.-V.

## Declaration of interests

The authors declare no competing interests.

## Supplemental information

Document S1. Figure S1

Table S1. mRNA index primers, related to step 26

Table S2. gDNA index primers, related to step 69

## References

[1] Kremer L.P.M., Braun M.M., Ovchinnikova S., Küchenhoff L., Cerrizuela S., Martin-Villalba A., Anders S. (2024). Analyzing single-cell bisulfite sequencing data with MethSCAn. Nat. Methods.

[2] Kremer L.P.M., Cerrizuela S., El-Sammak H., Al Shukairi M.E., Ellinger T., Straub J., Korkmaz A., Volk K., Brunken J., Kleber S. (2024). DNA methylation controls stemness of astrocytes in health and ischaemia. Nature.

[3] Clark, S. (2019). scNMT-seq protocol. 10.17504/protocols.io.4iiguce.

[4] Clark S.J., Argelaguet R., Kapourani C.-A., Stubbs T.M., Lee H.J., Alda-Catalinas C., Krueger F., Sanguinetti G., Kelsey G., Marioni J.C. (2018). scNMT-seq enables joint profiling of chromatin accessibility DNA methylation and transcription in single cells. Nat. Commun..

[5] Hagemann-Jensen M., Ziegenhain C., Chen P., Ramsköld D., Hendriks G.-J., Larsson A.J.M., Faridani O.R., Sandberg R. (2020). Single-cell RNA counting at allele and isoform resolution using Smart-seq3. Nat. Biotechnol..

[6] Hagemann-Jensen, M., Ziegenhain, C., Chen, P., Ramsköld, D., Hendriks, G.-J., Larsson, A.J.M., Faridani, O.R., and Sandberg, R. (2020). Smart-seq3 Protocol. 10.17504/protocols.io.bcq4ivyw. 32518404

[7] Cerrizuela S., Kaya O., Kremer L.P.M., Sarvari A., Ellinger T., Straub J., Brunken J., Sanz-Morejón A., Korkmaz A., Martín-Villalba A. (2022). High-throughput scNMT protocol for multiomics profiling of single cells from mouse brain and pancreatic organoids. STAR Protoc..

[8] Dimitriu M.A., Lazar-Contes I., Roszkowski M., Mansuy I.M. (2022). Single-Cell Multiomics Techniques: From Conception to Applications. Front. Cell Dev. Biol..

[9] Argelaguet R., Clark S.J., Mohammed H., Stapel L.C., Krueger C., Kapourani C.-A., Imaz-Rosshandler I., Lohoff T., Xiang Y., Hanna C.W. (2019). Multi-omics profiling of mouse gastrulation at single-cell resolution. Nature.

[10] Buenrostro J.D., Wu B., Litzenburger U.M., Ruff D., Gonzales M.L., Snyder M.P., Chang H.Y., Greenleaf W.J. (2015). Single-cell chromatin accessibility reveals principles of regulatory variation. Nature.

[11] Parekh S., Ziegenhain C., Vieth B., Enard W., Hellmann I. (2018). zUMIs - A fast and flexible pipeline to process RNA sequencing data with UMIs. GigaScience.

[12] Andrews S., Krueger F., Segonds-Pichon A., Biggins L., Krueger C., Wingett S. FastQC. (2010). A quality control tool for high throughput sequence data.

[13] Dobin A., Davis C.A., Schlesinger F., Drenkow J., Zaleski C., Jha S., Batut P., Chaisson M., Gingeras T.R. (2013). STAR: ultrafast universal RNA-seq aligner. Bioinformatics.

[14] Krueger, F. (2015). Trim Galore. A Wrapper Tool Around Cutadapt and FastQC to Consistently Apply Quality and Adapter Trimming to FastQ Files 516-517.

[15] Krueger F., Andrews S.R. (2011). Bismark: a flexible aligner and methylation caller for Bisulfite-Seq applications. Bioinformatics.

[16] Kremer L.P.M., Cerrizuela S., Dehler S., Stiehl T., Weinmann J., Abendroth H., Kleber S., Laure A., El Andari J., Anders S. (2021). High throughput screening of novel AAV capsids identifies variants for transduction of adult NSCs within the subventricular zone. Mol. Ther. Methods Clin. Dev..

[17] Wollny D., Zhao S., Everlien I., Lun X., Brunken J., Brüne D., Ziebell F., Tabansky I., Weichert W., Marciniak-Czochra A., Martin-Villalba A. (2016). Single-Cell Analysis Uncovers Clonal Acinar Cell Heterogeneity in the Adult Pancreas. Dev. Cell.

[18] Krieger T.G., Le Blanc S., Jabs J., Ten F.W., Ishaque N., Jechow K., Debnath O., Leonhardt C.-S., Giri A., Eils R. (2021). Single-cell analysis of patient-derived PDAC organoids reveals cell state heterogeneity and a conserved developmental hierarchy. Nat. Commun..

[19] Picelli S., Faridani O.R., Björklund A.K., Winberg G., Sagasser S., Sandberg R. (2014). Full-length RNA-seq from single cells using Smart-seq2. Nat. Protoc..

