# Supplementary material for: Protocol update to: High-throughput scNMT protocol for multiomics profiling of single cells from mouse brain and pancreatic organoids
Source: STAR Protoc. 2025 Jul 24;6(3):103980. doi: 10.1016/j.xpro.2025.103980 (PMC12311600; doi:10.1016/j.xpro.2025.103980)
Supplement: Document S1. Figure S1 [file mmc1.pdf]

Figure S1. gDNA library amplification indexes combination, related to step 69

Combinatorial indexing PE and iTAG index primers

PE (i5) iTAG

Row		Column 1	2	3	4
A	1	1	17	33	49
B	2	2	18	34	50
C	3	3	19	35	51
D	4	4	20	36	52
E	5	5	21	37	53
F	6	6	22	38	54
G	7	7	23	39	55
H	8	8	24	40	56
I	9	9	25	41	57
J	10	10	26	42	58
K	11	11	27	43	59
L	12	12	28	44	60
M	13	13	29	45	61
N	14	14	30	46	62
O	15	15	31	47	63
P	16	16	32	48	64

Row		5	6	7	8
A	17	65	81	1	17
B	18	66	82	2	18
C	19	67	83	3	19
D	20	68	84	4	20
E	21	69	85	5	21
F	22	70	86	6	22
G	23	71	87	7	23
H	24	72	88	8	24
I	25	73	89	9	25
J	26	74	90	10	26
K	27	75	91	11	27
L	28	76	92	12	28
M	29	77	93	13	29
N	30	78	94	14	30
O	31	79	95	15	31
P	32	80	96	16	32

Row		9	10	11	12
A	33	33	49	65	81
B	34	34	50	66	82
C	35	35	51	67	83
D	36	36	52	68	84
E	37	37	53	69	85
F	38	38	54	70	86
G	39	39	55	71	87
H	40	40	56	72	88
I	41	41	57	73	89
J	42	42	58	74	90
K	43	43	59	75	91
L	44	44	60	76	92
M	45	45	61	77	93
N	46	46	62	78	94
O	47	47	63	79	95
P	48	48	64	80	96

Row		13	14	15	16
A	49	1	17	33	49
B	50	2	18	34	50
C	51	3	19	35	51
D	52	4	20	36	52
E	53	5	21	37	53
F	54	6	22	38	54
G	55	7	23	39	55
H	56	8	24	40	56
I	57	9	25	41	57
J	58	10	26	42	58
K	59	11	27	43	59
L	60	12	28	44	60
M	61	13	29	45	61
N	62	14	30	46	62
O	63	15	31	47	63
P	64	16	32	48	64

Row		17	18	19	20
A	65	65	81	1	17
B	66	66	82	2	18
C	67	67	83	3	19
D	68	68	84	4	20
E	69	69	85	5	21
F	70	70	86	6	22
G	71	71	87	7	23
H	72	72	88	8	24
I	73	73	89	9	25
J	74	74	90	10	26
K	75	75	91	11	27
L	76	76	92	12	28
M	77	77	93	13	29
N	78	78	94	14	30
O	79	79	95	15	31
P	80	80	96	16	32

Row		21	22	23	24
A	81	33	49	65	81
B	82	34	50	66	82
C	83	35	51	67	83
D	84	36	52	68	84
E	85	37	53	69	85
F	86	38	54	70	86
G	87	39	55	71	87
H	88	40	56	72	88
I	89	41	57	73	89
J	90	42	58	74	90
K	91	43	59	75	91
L	92	44	60	76	92
M	93	45	61	77	93
N	94	46	62	78	94
O	95	47	63	79	95
P	96	48	64	80	96
